# The Role of Osteopontin (*OPN*/*SPP1*) Haplotypes in the Susceptibility to Crohn's Disease

**DOI:** 10.1371/journal.pone.0029309

**Published:** 2011-12-29

**Authors:** Jürgen Glas, Julia Seiderer, Corinna Bayrle, Martin Wetzke, Christoph Fries, Cornelia Tillack, Torsten Olszak, Florian Beigel, Christian Steib, Matthias Friedrich, Julia Diegelmann, Darina Czamara, Stephan Brand

**Affiliations:** 1 Department of Medicine II - Grosshadern, Ludwig-Maximilians-University, Munich, Germany; 2 Department of Preventive Dentistry and Periodontology, Ludwig-Maximilians-University, Munich, Germany; 3 Department of Human Genetics, Rheinisch-Westfälische Technische Hochschule (RWTH), Aachen, Germany; 4 Department of Pediatrics, Hannover Medical School, Hannover, Germany; 5 Division of Gastroenterology, Brigham and Women's Hospital, Harvard Medical School, Boston, Massachusetts, United States of America; 6 Max-Planck-Institute of Psychiatry, Munich, Germany; Ulm University, Germany

## Abstract

**Background:**

Osteopontin represents a multifunctional molecule playing a pivotal role in chronic inflammatory and autoimmune diseases. Its expression is increased in inflammatory bowel disease (IBD). The aim of our study was to analyze the association of osteopontin (*OPN*/*SPP1*) gene variants in a large cohort of IBD patients.

**Methodology/Principal Findings:**

Genomic DNA from 2819 Caucasian individuals (n = 841 patients with Crohn's disease (CD), n = 473 patients with ulcerative colitis (UC), and n = 1505 healthy unrelated controls) was analyzed for nine *OPN* SNPs (rs2728127, rs2853744, rs11730582, rs11739060, rs28357094, rs4754 = p.Asp80Asp, rs1126616 = p.Ala236Ala, rs1126772 and rs9138). Considering the important role of osteopontin in Th17-mediated diseases, we performed analysis for epistasis with IBD-associated *IL23R* variants and analyzed serum levels of the Th17 cytokine IL-22. For four *OPN* SNPs (rs4754, rs1126616, rs1126772 and rs9138), we observed significantly different distributions between male and female CD patients. rs4754 was protective in male CD patients (p = 0.0004, OR = 0.69). None of the other investigated *OPN* SNPs was associated with CD or UC susceptibility. However, several *OPN* haplotypes showed significant associations with CD susceptibility. The strongest association was found for a haplotype consisting of the 8 *OPN* SNPs rs2728127-rs2853744-rs11730582-rs11439060-rs28357094-rs112661-rs1126772-rs9138 (omnibus p-value = 2.07×10^−8^). Overall, the mean IL-22 secretion in the combined group of *OPN* minor allele carriers with CD was significantly lower than that of CD patients with *OPN* wildtype alleles (p = 3.66×10^−5^). There was evidence for weak epistasis between the *OPN* SNP rs28357094 with the *IL23R* SNP rs10489629 (p = 4.18×10^−2^) and between *OPN* SNP rs1126616 and *IL23R* SNP rs2201841 (p = 4.18×10^−2^) but none of these associations remained significant after Bonferroni correction.

**Conclusions/Significance:**

Our study identified *OPN* haplotypes as modifiers of CD susceptibility, while the combined effects of certain *OPN* variants may modulate IL-22 secretion.

## Introduction

The pathogenesis of inflammatory bowel diseases (IBD) such as Crohn's disease (CD) and ulcerative colitis (UC) is only partially understood. Currently, these diseases are assumed to be triggered by an exaggerated immune response to intestinal bacteria in a genetically susceptible host. In addition to the nucleotide-binding oligomerization domain 2/caspase recruitment domain-containing protein 15 (*NOD2/CARD15*) [1], [2], various novel susceptibility loci such as the interleukin-23 receptor (*IL23R*) [3], [4], the *ATG16L1* (autophagy-related 16-like 1) gene [5], [6] and variants in the 5p13.1 region [7] have been identified as susceptibility variants in CD patients. Based on new insights in the genetic background of CD, there is raising evidence for a key role of innate immunity and CD-related inflammatory pathways such as IL-23/IL-17 mediated T cell responses [8]. Recently, osteopontin (OPN, also known as Eta-1), an extracellular matrix glycosylated phosphoprotein produced by immune cells, epithelial cells and osteoblasts has been identified as an important molecule involved in tissue repair, inflammation and autoimmunity as well as tumour growth [9], [10], [11], [12]. So far, two forms of osteopontin have been identified - secreted osteopontin (sOPN) seems to be involved in the production of pathogenetic Th1 and Th17 cells, while an intracellular form of osteopontin (iOPN) is a key regulator for Toll like receptor-9 (TLR9) and/or TLR7-dependent interferon-α (IFN-α) expression by plasmacytoid dendritic cells (DCs) and Th17 development [13]. There is evidence for a key role of osteopontin in Th1- and Th17-mediated diseases [10], [14], [15] such as rheumatoid arthritis [16], [17], [18], psoriasis [19] and multiple sclerosis [20], [21], [22], [23]. In addition, osteopontin has also shown to be involved in granuloma formation [10], cell migration [24], [25], [26], and IL-12 production [27], [28], [29].

Osteopontin is expressed in the terminal ileum of CD patients [30] and seems to be closely involved in the Th1 immune response associated with CD [31], [32], [33], [34]. Moreover, it has also been reported to play an important role in the pathogenesis of UC [35], [36], [37], [38]. Analyzing the exact role of osteopontin in a murine model of acute colitis, a recent study demonstrated that *Opn^−/−^* mice showed increased serum levels of TNF-α but also reduced mRNA expression of IL-1β and matrix metalloproteinases as well as decreased blood levels of IL-22 [39]. In contrast, in a chronic DSS model, *Opn^−/−^* mice were protected from mucosal inflammation showing lower serum IL-12 levels compared to wildtype mice and neutralization of *OPN* in wildtype mice abrogated colitis [39]. These findings implicate a dual function of osteopontin in intestinal inflammation characterized by activation of innate immunity and Th17 cytokines such as IL-22 initiating mucosal repair in acute inflammation; while under conditions of chronic intestinal inflammation it may promote the Th1 response and thereby enhancing inflammation [39]. Further investigations by daSilva et al. in a DSS model demonstrated that osteopontin administration reduced the disease activity index, improved red blood cell counts, and reduced gut neutrophil activity compared with the DSS-treated wildtype mice [37]. Interestingly, the study by Heilmann et al. demonstrated a significant correlation of osteopontin serum levels with disease activity in human CD [39].

In this study, we aimed to analyze the role of *OPN* gene variants on IBD disease susceptibility and phenotype. We also investigated for potential epistasis with IBD-associated *IL23R* gene variants. In total, we genotyped nine common single nucleotide polymorphisms (SNPs) in the *OPN* gene, which were previously shown to be associated with other immune-mediated diseases [40], [41], [42], [43]. Last, based on the important role demonstrated for IL-22 in colitis experiments in *Opn−/−* mice [39], we analyzed the effect of *OPN* gene variants on IL-22 serum levels.

## Methods

### Ethics statement

Written, informed consent was obtained from all patients prior to inclusion into the study. In the case of minors, the consent was provided by the parents. This study was approved by the Ethics committee of the Medical Faculty of Ludwig-Maximilians-University Munich. The study protocol adhered to the ethical principles for medical research involving human subjects of the Helsinki Declaration (as described in detail under: http://www.wma.net/en/30publications/10policies/b3/index.html).

### Study population

Our study population comprised 2819 individuals of Caucasian origin including n = 841 patients with CD, n = 473 patients with UC and n = 1505 healthy unrelated controls. All phenotypic data were collected blind to the results of genotyping and included detailed demographic and clinical parameters (disease behaviour, anatomic manifestation of IBD, complications, surgical or immunosuppressive therapy). The diagnosis of CD and UC was based on established guidelines according to endoscopic, radiological, and histopathological parameters. For classification of CD patients, the Montreal classification [44] based on age at diagnosis (A), location (L), and behaviour (B) of disease was used. In patients with UC, anatomic location was also based on the Montreal classification, based on the criteria ulcerative proctitis (E1), left-sided UC (distal UC; E2), and extensive UC (pancolitis; E3). Patients with indeterminate colitis were excluded from the study. The clinical characteristics of the IBD study population are shown in Table 1.

**Table 1:** Demographic characteristics of the IBD study population.

	Crohn's disease	Ulcerative colitis	Controls
	*n = 841*	*n = 473*	*n = 1505*
**Gender**			
Male (%)	49.2	47.3	62.6
Female (%)	50.8	52.7	37.4
**Age** (yrs)			
Mean ± SD	39.4±13.1	41.7±14.4	45.9±10.7
Range	10–80	7–85	18–71
**Body mass index**			
Mean ± SD	23.1±4.2	23.9±4.1	
Range	13–40	15–41	
**Age at diagnosis**			
(yrs)			
Mean ± SD	27.9±11.7	31.9±13.4	
Range	7–71	9–81	
**Disease duration**			
(yrs)			
Mean ± SD	12.2±8.4	11.0±7.7	
Range	0–44	1–40	
**Positive family**			
**history of IBD (%)**	16.1	16.0	

### DNA extraction

From all study participants, blood samples were taken and genomic DNA was isolated from peripheral blood leukocytes using the DNA blood mini kit from Qiagen (Hilden, Germany) according to the manufacturer's guidelines.

### Genotyping of *OPN* gene variants

Nine *OPN* SNPs (rs2728127, rs2853744, rs11730582, rs11739060, rs28357094, rs4754 = p.Asp80Asp, rs1126616 = p.Ala236Ala, rs1126772 and rs9138) were genotyped by PCR and melting curve analysis using a pair of fluorescence resonance energy transfer (FRET) probes in a LightCycler® 480 Instrument (Roche Diagnostics, Mannheim, Germany) as previously described in detail [45], [46], [47], [48]. The selection of these SNPs was based on previous studies in which associations for several of these *OPN* variants with autoimmune and Th1- and Th17-mediated diseases have been shown [40], [41], [42], [43], [49], [50], [51], [52], [53]. The donor fluorescent molecule (fluorescein) at the 3′-end of the sensor probe (or the anchor probe in the case of rs2853744 and rs11730582) is excited at its specific fluorescence excitation wavelength (533 nm) and the energy is transferred to the acceptor fluorescent molecule at the 5′-end (LightCycler Red 610, 640 or 670) of the anchor probe (or the sensor probe in the case of rs2853744 and rs11730582). The specific fluorescence signal emitted by the acceptor molecule is detected by the optical unit of the LightCycler. The sensor probe is exactly matching to one allele of each SNP, preferentially to the rarer allele, whereas in the case of the other allele, there is a mismatch resulting in a lower melting temperature. The total volume of the PCR was 5 µl containing 25 ng of genomic DNA, 1× Light Cycler 480 Genotyping Master (Roche Diagnostics), 2.5 pmol of each primer and 0.75 pmol of each FRET probe (TIB MOLBIOL, Berlin, Germany). In the case of rs11739060, the concentration of the forward primer, and in the case of rs1126772, the concentration of the reverse primer was reduced to 0.5 pmol. The PCR comprised an initial denaturation step (95°C for 10 min) and 45 cycles (95°C for 10 sec, primer annealing temperature as given in the Supplementary data (Table S1) for 10 sec, 72°C for 15 sec). The melting curve analysis comprised an initial denaturation step (95°C for 1 min), a step rapidly lowering the temperature to 40°C and holding for 2 min, and a heating step slowly (1 acquisition/°C) increasing the temperature up to 95°C and continuously measuring the fluorescence intensity. The results of the melting curve analysis have been confirmed by analyzing two patient samples for each possible genotype using sequence analysis. For sequencing, the total volume of the PCR was 100 µl containing 250 ng of genomic DNA, 1× PCR buffer (Qiagen, Hilden, Germany), a final MgCl_2_ concentration of 2 mM, 0.5 mM of a dNTP mix (Sigma, Steinheim, Germany), 2.5 units of HotStar Plus Taq™ DNA polymerase (Qiagen) and 10 pmol of each primer (TIB MOLBIOL). The PCR comprised an initial denaturation step (95°C for 5 min), 35 cycles (denaturation at 94°C for 30 sec, primer annealing at 60°C for 30 sec, extension at 72°C for 30 sec) and a final extension step (72°C for 10 min). The PCR products were purified using the QIAquick PCR Purification Kit (Qiagen) and sequenced by a commercial sequencing company (Sequiserve, Vaterstetten, Germany). All sequences of primers and FRET probes and primer annealing temperatures used for genotyping and for sequence analysis are given in Tables S1 and S2.

### Genotyping of *IL23R* gene variants

Genotypes of 10 IBD-associated *IL23R* gene variants (rs1004819, rs7517847, rs10489629, rs2201841, rs11465804, rs11209026 = p.Arg381Gln, rs1343151, rs10889677, rs11209032, rs1495965) were available for all study patients and controls from previous studies [4], [54].

### Analysis of IL-22 serum levels in CD patients

In order to investigate a potential correlation between IL-22 serum expression and *OPN/SPP1* genotype, IL-22 serum levels were determined in a subcohort of CD patients, in which serum samples and genomic DNA was available. IL-22 serum levels for the majority of these patients were available from a previous study [55]. For the ELISA analysis, the human IL-22 Quantikine Elisa Kit (R&D Systems, Minneapolis, MN) was used following the manufacturer's guidelines. The following steps were performed: First, all reagents, working standards, and samples were prepared as outlined in the manufacturer's guidelines. Next, 100 µL of assay diluent RD1-88 were added to each well. After this step, 100 µL of standard, control, or sample were added per well and incubated for two hours at room temperature. Then, each well was aspirated and washed four times. 200 µL of a mouse monoclonal antibody against IL-22 conjugated to horseradish peroxidase were added and the plates were incubated for two hours at room temperature. After this, wells were aspirated and washed four times. Next, 200 µL of substrate solution were added to each well. The plates were incubated for 30 minutes at room temperature to allow colour development while being protected from light. Next, 50 µL of stop solution were added to each well and the optical density of each well was determined within 30 minutes, using a microplate reader set to 450 nm. IL-22 serum levels (pg/ml) were calculated from a standard curve of known IL-22 concentrations.

### Statistical analyses

Each genetic marker was tested for Hardy-Weinberg equilibrium in the control population. Single-marker allelic tests were performed with Fisher's exact test. All tests were two-tailed, considering p-values<0.05 as significant. Odds ratios were calculated for the minor allele at each SNP. For multiple comparisons, Bonferroni correction was applied where indicated. rs4754 deviated from the Hardy-Weinberg equilibrium in the control population (p = 0.0005) and was therefore excluded from the haplotype analysis. Haplotype analysis was conducted with PLINK (http://pngu.mgh.harvard.edu/~purcell/plink/) and the –hap-logistic option using a sliding-window approach with 2 up to 8 included SNPs. Interaction between different polymorphisms were also tested with PLINK and the –epistasis command. For analyzing potential differences of IL-22 serum levels between the carriers of the different *OPN* gene variants, the mean IL-22 serum level of carriers of the wildtype allele of each SNP was compared with the mean IL-22 serum level of carriers of the minor allele ( = combined group of heterozygous and homozygous carriers) using Student's t-test.

## Results

### Frequency distribution of *OPN* gene variants and their role in IBD susceptibility

For all three subgroups (CD, UC, and controls), the minor allele frequencies of the nine *OPN* SNPs (rs2728127, rs2853744, rs11730582, rs11739060, rs28357094, rs4754 = p.Asp80Asp, rs1126616 = p.Ala236Ala, rs1126772 and rs9138) are summarized in Table 2. With the exception of rs4754, no significant differences in the allele frequencies were observed comparing CD and UC patients to healthy controls (Table 2). Our analysis revealed a weak association of SNP rs4754 (p.Asp80Asp) with CD susceptibility (p = 1.28×10^−2^; OR (95% CI) 0.85 [0.74–0.96]). Similar to CD, rs4754 (p.Asp80Asp) decreased susceptibility to UC, although this association did not reach significance in univariate analysis (p = 5.25×10^−2^; OR (95% CI) 0.85 [0.70–1.00]) (Table 2). Moreover, both associations of rs4754 (regarding CD and UC susceptibility) were not statistically significant after Bonferroni correction, suggesting that these *OPN* variants are not major contributors to IBD susceptibility on their own. In addition, rs4754 deviated from the Hardy-Weinberg equilibrium in the control population (p = 0.0005) and was therefore excluded from the haplotype analysis. However, several OPN haplotypes were associated with CD susceptibility. As shown in table 3, the strongest association was found for a haplotype consisting of the 8 *OPN* SNPs rs2728127-rs2853744-rs11730582-rs11439060-rs28357094-rs112661-rs1126772-rs9138 with an omnibus p-value of 2.07×10^−8^ (Table 3); if rs4754 would be included into this haplotype block, the omnibus p-value would increase further to p = 3.67×10^−12^. In contrast, there were no associations of certain *OPN* haplotypes with UC susceptibility (Table 4).

**Table 2:** Associations of *OPN/SPP1* gene markers in CD and UC case-control association studies.

Cohort		Crohn's disease			Ulcerative colitis			Controls
Number of individuals		n = 841			n = 473			n = 1505
Gene marker	Minor allele	MAF	p value	OR [95% CI]	MAF	p value	OR [95% CI]	MAF
rs2728127	G	0.295	0.841	0.98 [0.86–1.12]	0.274	0.162	0.89 [0.75–1.05]	0.298
rs2853744	T	0.071	0.520	0.92 [0.73–1.16]	0.080	0.725	1.05 [0.80–1.38]	0.076
rs11730582	C	0.503	0.125	1.09 [0.97–1.24]	0.495	0.430	1.06 [0.92–1.23]	0.479
rs11739060	insG	0.290	0.815	0.98 [0.86–1.12]	0.274	0.266	0.91 [0.77–1.07]	0.294
rs28357094	G	0.223	0.437	1.06 [0.92–1.23]	0.198	0.358	0.91 [0.76–1.10]	0.213
rs4754 = p.Asp80Asp	C	0.281	**0.013**	**0.85 [0.74–0.96]**	0.282	0.053	0.85 [0.70–1.00]	0.316
rs1126616 = p.Ala236Ala	T	0.279	0.892	0.99 [087–1.13]	0.285	0.804	0.97 [0.82–1.14]	0.281
rs1126772	G	0.220	0.852	1.01 [0.87–1.17]	0.213	0.783	0.97 [0.81–1.17]	0.218
rs9138	C	0.278	0.919	1.01 [0.88–1.15]	0.280	0.868	1.02 [0.86–1.20]	0.276

Minor allele frequencies (MAF), allelic test *P*-values, and odds ratios (OR, shown for the minor allele) with 95% confidence intervals (CI) are depicted for both the CD and UC case-control cohorts. rs4754 deviated from the Hardy-Weinberg equilibrium (HWE) in the control population (p = 0.0005) and was therefore excluded from further analysis.

**Table 3:** Haplotypes of *OPN* SNPs in Crohn's disease (CD) case-control sample (846 cases and 1510 controls) and omnibus p-values for association with CD susceptibility.

Haplotype combination	Omnibus p-value
rs2728127-rs2853744	9.09×10^−1^
rs2853744-rs11730582	2.74×10^−1^
rs11730582-rs11439060	6.87×10^−2^
rs11439060-rs28357094	2.25×10^−1^
rs28357094-rs1126616	6.11×10^−1^
rs1126616-rs1126772	1.81×10^−1^
rs1126772-rs9138	4.71×10^−1^
rs2728127-rs2853744-rs11730582	1.95×10^−1^
rs2853744-rs11730582-rs11439060	1.34×10^−1^
rs11730582-rs11439060-rs28357094	5.37×10^−2^
rs11439060-rs28357094-rs1126616	2.72×10^−1^
rs28357094-rs1126616-rs1126772	3.72×10^−1^
rs1126616-rs1126772-rs9138	6.45×10^−1^
rs2728127-rs2853744-rs11730582-rs11439060	**2.15×10^−2^**
rs2853744-rs11730582-rs11439060-rs28357094	1.62×10^−1^
rs11730582-rs11439060-rs28357094-rs1126616	1.35×10^−1^
rs11439060-rs28357094-rs1126616-rs1126772	2.74×10^−1^
rs28357094-rs1126616-rs1126772-rs9138	6.77×10^−1^
rs2728127-rs2853744-rs11730582-rs11439060-rs28357094	**3.77×10^−2^**
rs2853744-rs11730582-rs11439060-rs28357094-rs1126616	1.98×10^−1^
rs11730582-rs11439060-rs28357094-rs1126616-rs1126772	6.95×10^−2^
rs11439060-rs28357094-rs1126616-rs1126772-rs9138	3.84×10^−1^
rs2728127-rs2853744-rs11730582-rs11439060-rs28357094-rs112661	5.03×10^−2^
rs2853744-rs11730582-rs11439060-rs28357094-rs1126616-rs1126772	6.86×10^−2^
rs11730582-rs11439060-rs28357094-rs1126616-rs1126772-rs9138	5.75×10^−2^
rs2728127-rs2853744-rs11730582-rs11439060-rs28357094-rs112661-rs1126772	**1.44×10^−7^**
rs2853744-rs11730582-rs11439060-rs28357094-rs1126616-rs1126772-rs9138	**2.76×10^−5^**
rs2728127-rs2853744-rs11730582-rs11439060-rs28357094-rs112661-rs1126772-rs9138	**2.07×10^−8^**

Significant p-values<0.05 are depicted in bold. All significant p-values remained significant after 10.000 permutations.

**Table 4:** Haplotypes of *OPN* SNPs in ulcerative colitis (UC) case-control sample (501 cases and 1510 controls) and omnibus p-values for association with UC susceptibility.

Haplotype combination	Omnibus p-value
rs2728127-rs2853744	5.62×10^−1^
rs2853744-rs11730582	3.72×10^−1^
rs11730582-rs11439060	7.01×10^−1^
rs11439060-rs28357094	9.54×10^−1^
rs28357094-rs1126616	8.08×10^−1^
rs1126616-rs1126772	2.80×10^−1^
rs1126772-rs9138	2.65×10^−1^
rs2728127-rs2853744-rs11730582	5.24×10^−1^
rs2853744-rs11730582-rs11439060	6.86×10^−1^
rs11730582-rs11439060-rs28357094	8.62×10^−1^
rs11439060-rs28357094-rs1126616	7.28×10^−1^
rs28357094-rs1126616-rs1126772	3.86×10^−1^
rs1126616-rs1126772-rs9138	3.02×10^−1^
rs2728127-rs2853744-rs11730582-rs11439060	8.26×10^−1^
rs2853744-rs11730582-rs11439060-rs28357094	4.98×10^−1^
rs11730582-rs11439060-rs28357094-rs1126616	8.39×10^−1^
rs11439060-rs28357094-rs1126616-rs1126772	1.97×10^−1^
rs28357094-rs1126616-rs1126772-rs9138	5.24×10^−1^
rs2728127-rs2853744-rs11730582-rs11439060-rs28357094	5.02×10^−1^
rs2853744-rs11730582-rs11439060-rs28357094-rs1126616	8.25×10^−1^
rs11730582-rs11439060-rs28357094-rs1126616-rs1126772	5.07×10^−1^
rs11439060-rs28357094-rs1126616-rs1126772-rs9138	3.01×10^−1^
rs2728127-rs2853744-rs11730582-rs11439060-rs28357094-rs112661	7.27×10^−1^
rs2853744-rs11730582-rs11439060-rs28357094-rs1126616-rs1126772	5.85×10^−1^
rs11730582-rs11439060-rs28357094-rs1126616-rs1126772-rs9138	5.36×10^−1^
rs2728127-rs2853744-rs11730582-rs11439060-rs28357094-rs112661-rs1126772	5.86×10^−1^
rs2853744-rs11730582-rs11439060-rs28357094-rs1126616-rs1126772-rs9138	5.95×10^−1^
rs2728127-rs2853744-rs11730582-rs11439060-rs28357094-rs112661-rs1126772-rs9138	5.00×10^−1^

None of the haplotypes was significantly associated with UC susceptibility (p>0.05).

### Analysis for gender-specific differences in *OPN* variants

Previous studies demonstrated significant gender-specific effects of *OPN* variants in systemic lupus erythematosus (SLE) and type 1-diabetes, particularly in male patients [43], [50]. Considering the deviation of rs4754 from the Hardy-Weinberg equilibrium, we therefore investigated potential gender-specific effects in IBD susceptibility. For four *OPN* SNPs (rs4754, rs1126616, rs1126772 and rs9138), we observed significantly different distributions between male and female CD patients. Interestingly, for these SNPs, there was an opposite direction of the association results for males and females (rs4754: p = 0.0004, OR = 0.69 [95% CI: 0.56–0.85] (males), p = 0.7693, OR = 1.03 (females); rs1126616: p = 0.1187, OR = 0.85 (males), p = 0.2676, OR = 1.12 (females); rs1126772: p = 0.1679, OR = 0.85 (males), p = 0.0893, OR = 1.21 (females); rs9138: p = 0.1256, OR = 0.85 (males), p = 0.0864, OR = 1.19 (females)). Given that the most pronounced difference between male and female CD patients was found for rs4754, which deviated from the Hardy-Weinberg equilibrium in the control population, we next investigated if the deviation from Hardy-Weinberg equilibrium is based on a gender-specific effect. This analysis revealed that there was significant deviation from Hardy-Weinberg equilibrium in male controls (n = 917; p = 0.0018), but not in female controls (n = 547; p = 0.1347), confirming the gender-specific effect of this *OPN* SNP found in CD patients.

### Analysis for epistasis between *OPN* variants and *IL23R* variants

To investigate if *OPN* variants modify IBD susceptibility by epistastic interaction with other Th17-related IBD susceptibility genes, we next analyzed for potential epistasis of *OPN* variants with main IBD-associated *IL23R* variants. We found evidence of weak epistasis between the *OPN* SNP rs28357094 with the *IL23R* SNP rs10489629 (p = 4.18×10^−2^) and between *OPN* SNP rs1126616 and *IL23R* SNP rs2201841 (p = 4.18×10^−2^) but none of these associations remained significant after Bonferroni correction (Table 5).

**Table 5:** Analysis for epistatic interactions between *OPN* SNPs and *IL23R* SNPs regarding CD susceptibility (based on 1510 controls and 704 cases).

*OPN* SNPs	rs2728127	rs2853744	rs11730582	rs11439060	rs28357094	rs1126616	rs1126772	rs9138
***IL23R*** ** SNPs**								
**rs1004819**	5.45×10^−1^	1.34×10^−1^	3.00×10^−1^	8.83×10^−1^	5.93×10^−1^	3.69×10^−1^	2.86×10^−1^	4.52×10^−1^
**rs7517847**	4.52×10^−1^	7.94×10^−1^	2.53×10^−1^	5.96×10^−1^	3.98×10^−1^	8.57×10^−1^	4.97×10^−1^	5.79×10^−1^
**rs10489629**	1.90×10^−1^	3.31×10^−1^	5.54×10^−1^	2.32×10^−1^	**4.18×10^−2^**	8.05×10^−1^	4.31×10^−1^	6.28×10^−1^
**rs2201841**	2.49×10^−1^	2.18×10^−1^	2.43×10^−1^	1.74×10^−1^	5.91×10^−2^	**4.71×10^−2^**	6.46×10^−2^	8.10×10^−2^
**rs11465804**	8.02×10^−1^	5.97×10^−1^	5.98×10^−1^	7.45×10^−1^	9.86×10^−2^	6.19×10^−1^	4.54×10^−1^	6.18×10^−1^
**rs11209026 = **	6.71×10^−1^	8.056×10^−1^	2.466×10^−1^	6.64×10^−1^	5.17×10^−1^	8.87×10^−1^	6.29×10^−1^	9.76×10^−1^
**p.Arg381Gln**								
**rs1343151**	6.65×10^−1^	2.25×10^−1^	9.68×10^−1^	7.34×10^−1^	1.23×10^−1^	9.98×10^−1^	3.32×10^−1^	8.79×10^−1^
**rs10889677**	2.49×10^−1^	3.09×10^−1^	3.29×10^−1^	1.53×10^−1^	6.05×10^−2^	5.88×10^−2^	8.51×10^−2^	9.73×10^−2^
**rs11209032**	4.46×10^−1^	2.92×10^−1^	2.71×10^−1^	3.58×10^−1^	2.71×10^−1^	1.91×10^−1^	3.46×10^−1^	3.75×10^−1^
**rs1495965**	1.79×10^−1^	2.77×10^−1^	9.52×10^−2^	1.34×10^−1^	1.11×10^−1^	1.94×10^−1^	2.82×10^−1^	2.39×10^−1^

Significant p-values<0.05 are depicted in bold. However, these associations did not remain significant after Bonferroni correction.

### Correlation between *OPN* variants and IL-22 serum levels in CD patients

Based on the recent data of Heilmann et al. [39] demonstrating decreased blood levels of IL-22 in acute colitis in *Opn−/−* mice, we next investigated a potential association of *OPN* variants and IL-22 serum levels in a subcohort of CD patients. No correlation was found between *OPN* SNPs and IL-22 serum levels (Table 6). However, overall the IL-22 serum levels tended to be lower in the carriers of *OPN* minor alleles, which was statistically significant when the mean IL-22 expression level of carriers of the 9 investigated *OPN* SNPs minor alleles (homo- and heterozygous carriers) were compared to the homozygous carriers of the wildtype allele (p = 3.6×10^−5^). Interestingly, for 7 out of 8 *OPN* SNPs forming the haplotype rs2728127-rs2853744-rs11730582-rs11439060-rs28357094-rs112661-rs1126772-rs9138, which was strongly associated with CD susceptibility (omnibus p-value 2.07×10^−8^), the IL-22 serum levels were nominally lower in CD carriers of the minor allele than in wildtype carriers, although these differences were for each SNP only small and statistically not significant (Table 6).

**Table 6:** *OPN* gene variants modulate IL-22 serum levels in CD patients.

*OPN* SNP	IL-22 serum levels	IL-22 serum levels	p-value
	in *OPN* wildtype	in *OPN* minor allele	
	carriers [pg/ml]	carriers* [pg/ml]	
rs2728127	39.72	37.28	0.537
rs2853744	38.24	39.54	0.854
rs11730582	42.07	37.18	0.341
rs11439060	39.72	37.28	0.537
rs28357094	39.23	37.36	0.614
rs4754 = p.Asp80Asp	40.19	36.78	0.380
rs1126616 = p.Ala236Ala	40.19	36.59	0.357
rs1126772	41.04	34.96	0.106
rs9138	40.35	36.59	0.333
**Mean**	**40.08**	**37.06**	**3.66×10^−5^**

The mean IL-22 serum level was analyzed for each *OPN* variant in a subgroup of 151 CD patients for which DNA for genotyping and serum for ELISA analysis was available. *P* values are given for the comparison of the mean IL-22 serum levels of carriers of the minor allele (*homozygous and heterozygous) compared to cytokine levels in homozygous wild-type carriers.

## Discussion

The presented study represents the first detailed analysis of *OPN* gene variants in IBD patients. In this study, there were no significant associations of single *OPN* SNPs with CD or UC susceptibility after Bonferroni correction for multiple testing; however, several *OPN* haplotypes were associated with CD susceptibility. The strongest association was found for a haplotype consisting of the 8 *OPN* SNPs (rs2728127-rs2853744-rs11730582-rs11439060-rs28357094-rs112661-rs1126772-rs9138; omnibus p-value 2.07×10^−8^). However, considering the strength of the association signals found for a number of other recently identified IBD susceptibility genes [56], [57], this argues against a major role for *OPN* in the genetic susceptibility for IBD. Given the strong association of osteopontin with Th1- and Th17-mediated diseases, the finding of an association of *OPN* haplotypes with CD, a Th1- and Th17-mediated disease, but not UC susceptibility is not surprising. In contrast, UC has been associated with a predominantly modified Th2 response but partially also with a Th17 immune response. The results of our haplotype analysis suggest that certain rare haplotypes significantly contribute to the genetic risk of CD. This is in agreement with recent results of the International IBD Genetics Consortium which identified a total of 71 CD susceptibility loci [56]. These 71 susceptibility loci explain only slightly more than 20% of CD heritability. Therefore, it is assumed that a number of rare SNPs and haplotypes contribute to the overall CD risk such as recently shown by us for *PXR* gene variants [58]. In addition, most likely a high number of common CD risk genes with small effect size are still unidentified but for their identification very large cohorts would be required.

So far, genetic variants in the *OPN* gene have shown to be involved in susceptibility to other immune-mediated diseases such as SLE [59], [60], oligoarticular juvenile idiopathic arthritis [61] and sarcoidosis [51]. Despite promising functional data, previous genotype analyses could not confirm *OPN* as significant disease-modifying gene in classical Th17-mediated diseases such as multiple sclerosis [62], [63] and rheumatoid arthritis [64]. Investigating the role of *OPN* as a susceptibility gene in SLE, a recent study demonstrated a significant association in male patients [50] – a phenomenon also seen in a study investigating *OPN* variants in type-1 diabetes, implicating a potential gender-specific mechanism acting in the autoimmune process [43]. Similarly, our analysis demonstrated gender-specific effects for four *OPN* SNPs, particularly for rs4754 which deviated from the Hardy-Weinberg equilibrium in male controls. Moreover, there was a significant association of this SNP with CD in male but not in female patients.

While osteopontin is closely involved in the Th1- and Th17-mediated immune response associated with CD [31], [32], [33], [34], its role in murine colitis models is controversially discussed. In one study, osteopontin deficiency protected mice from DSS-induced colitis [38], while in another study, osteopontin administration in *Opn−/−* mice reduced the disease activity index, improved red blood cell counts, and reduced gut neutrophil activity compared with the DSS-treated wildtype mice [37]. Interestingly, a recent study demonstrated that *Opn^−/−^* mice showed decreased blood levels of IL-22 [39]. Since we recently demonstrated that IL-22 serum levels are increased in CD and correlate with disease activity and the *IL23R* genotype [55], we next analyzed a potential association between *OPN* genotypes and IL-22 serum levels in CD patients. Overall, we observed lower IL-22 serum levels in the carriers of *OPN* minor alleles (homo- and heterozygous carriers), which was statistically significant when the mean IL-22 expression level of carriers of the 9 investigated *OPN* SNPs minor alleles was compared to the mean IL-22 serum level of the carriers of the homozygous wildtype alleles (p = 3.6×10^−5^). In 7 out of 8 *OPN/SPP1* SNPs forming the haplotype rs2728127-rs2853744-rs11730582-rs11439060-rs28357094-rs112661-rs1126772-rs9138, which was strongly associated with CD susceptibility, the IL-22 serum levels were nominally lower in CD carriers of the minor allele than in homozygous wildtype carriers, although these differences were for each SNP only small and statistically not significant. Similarly, there were no significant associations with CD or UC susceptibility with single *OPN* SNPs after Bonferroni correction, suggesting that only the combined effect of certain *OPN* SNPs and haplotypes leads to decreased basal IL-22 levels and increased CD susceptibility. We therefore hypothesize that certain *OPN* variants may increase the CD risk via decreased basal expression of IL-22, for which we and others demonstrated strong epithelial-protective properties [65], [66], [67], [68]. However, given the multitude of functions mediated by osteopontin, other disease-modulating properties of *OPN* haplotypes are likely and need further functional investigation.

In addition to increased wound healing, IL-22 mediates also early host defense against attaching and effacing bacterial pathogens [69], [70]. In line with the data of Heilmann et al. [39] demonstrating a dual role of osteopontin in intestinal inflammation, one might therefore hypothesize that carriers of *OPN* minor alleles with lower IL-22 serum levels are at high risk of developing intestinal inflammation due to the lack of IL-22-induced mucosal protection. Interestingly, *Opn−/−* mice demonstrated altered wound healing [71], which may be also related to decreased expression of IL-22, which is a strong enhancer of intestinal wound healing [65].

Recent studies in mice showed that osteopontin is involved in Th17 cell differentiation [72] and *Opn*-expressing DCs induce IL-17 production in T cells [21]. On the other hand, osteopontin expression in DCs is repressed by IFN-α and IFN-γ [73], [74]. This decreased osteopontin expression is associated with high production of IL-27, a Th17 cell-inhibiting cytokine that favors regulatory T cell development [75]. We recently demonstrated that IL-27 is also a protective factor for the intestinal epithelial barrier [76]. IL-27 induces anti-inflammatory and antibacterial responses in intestinal epithelial cells and increases cell restitution after wounding [76]. In mice with *Opn*-deficient DCs, substantially elevated levels of IL-27 are produced and *Opn* ^−/−^ mice develop delayed experimental autoimmune encephalitis with a Th1 rather than Th17-dominated response [73]. *Opn^−/−^* mice display a stronger Th1-mediated proinflammatory response during chronic inflammation while a reduced Th17 response during acute colitis protects them from mucosal inflammation [39], further strengthening the dual role of osteopontin in intestinal inflammation.

In summary, our study identified certain *OPN* haplotypes to be associated with CD susceptibility. *OPN* variants may modulate IL-22 secretion which is consistent with data in *Opn−/−* mice, in which low levels of the epithelial-protective cytokine IL-22 predispose to intestinal inflammation. However, the rather weak association signals found in this study argue against a significant role for *OPN* as major IBD susceptibility gene which is consistent with the recent IBD meta-analyses [56], [57]. Further functional analysis of large cohorts and detailed fine mapping is required to clarify the role of *OPN* variants in the genetic susceptibility to IBD.

## Supporting Information

Table S1 **Primer sequences, FRET probe sequences, and primer annealing temperatures used for genotyping** ***OPN*** **variants.**
(DOC)

Table S2 **Primer sequences used for the sequence analysis of** ***OPN*** **variants.**
(DOC)
