# Supplementary material for: The Role of Osteopontin (OPN/SPP1) Haplotypes in the Susceptibility to Crohn's Disease
Source: PLoS One. 2011 Dec 29;6(12):e29309. doi: 10.1371/journal.pone.0029309 (PMC3248444; doi:10.1371/journal.pone.0029309)
Supplement: Table S1 — Primer sequences, FRET probe sequences, and primer annealing temperatures used for genotyping OPN variants. (DOC) [file pone.0029309.s001.doc]

**Supplemental Table S1**

| **Polymorphism** | **Primer sequences** | **Primer annealing** | **FRET probe sequences** |
| --- | --- | --- | --- |
| rs2728127 | GGCTCCAGCATAATCTATTCCTA  TGGTTTGTAGGTAATAAGCACCCAT | 59 °C | GTTTTTAGAATTTTCAGACTTCCC-FL  LC670-CCACTAAATTGACAACATGACACGCTT |
| rs2853744 | AGCAGTGACACAGCGGAA  GAAGGCTATTGTTCAAGCCTACA | 56 °C | LC610-TCATCCTGCTCTCAGTCAGAAAC CTCCTGAAGCAGCCCTCTCAAGC-FL |
| rs11730582 | CATGGATGAGGGAACAAGGATA  GAAGGCTATTGTTCAAGCCTACA | 56 °C | LC640-AGGCAAGTTCTCTGAACTCCTT  TCACAAAGCTAAGCTTGAGTAGTAAAGGAC-FL |
| rs11739060 | CTGAATGCCCATCCCGTA  AAGCCCTCCCAGAATTTAAATGC | 60 °C | GTTTTTGGTTTTTTTTTGTTTTAACC-FL  LC640-AACCAGAGGGGGAAGTGTGGGAGC |
| rs28357094 | CTGAATGCCCATCCCGTA  TGACAACCAAGCCCTCCCAGAA | 60 °C | ATGACACAATCTCGCCGCC-FL  LC610-CCCTGTGTTGGTGGAGGATGTCTGC |
| rs4754=p.Asp80Asp | TAATTTTCAGACCCTTCCAAG  GTGAGACTCATCAGACTGGTGAGAA | 57 °C | AAGATGATGACGACCATGTGG-FL  LC610-CAGCCAGGACTCCATTGACTCGAA |
| rs1126616=p.Ala236Ala | TGAAACCCACAGCCACAAG  ATGGCTGTGGAATTCACGG | 58 °C | GGAAAGCCAATGATGAGAGCA-FL  LC640-TGAGCATTCCGATGTGATTGATAGTCA |
| rs1126772 | CATGAATTAGATAGTGCATCTTCTGAGG  TGTTATATTCTCTTTTTAAGTGGGTA | 58 °C | AAACACATCAGTTATTTCCAGAC-FL  LC640-CAAATAGATACACATTCAACCAATAAACTG |
| rs9138 | GTCTATGTTCATTCTATAGAAGAAATGC  TGTTATATTCTCTTTTTAAGTGGGTA | 58 °C | TGTTTGCGTCTACATAAATTTC-FL  LC610-TTCATGAGAGAATAACAAATATTAAAATACAGTG |

**Supplemental Table S1. Primer sequences, FRET probe sequences, and primer annealing temperatures used for genotyping *OPN* variants.** Note: FL: Fluorescein, LC610: LightCycler-Red 610; LC640: LightCycler-Red 640; LC670: LightCycler-Red 670. The polymorphic position within the sensor probe is underlined. A phosphate is linked to the 3'-end of the acceptor probe to prevent elongation by the DNA polymerase in the PCR
