# Supplementary material for: The Role of Osteopontin (OPN/SPP1) Haplotypes in the Susceptibility to Crohn's Disease
Source: PLoS One. 2011 Dec 29;6(12):e29309. doi: 10.1371/journal.pone.0029309 (PMC3248444; doi:10.1371/journal.pone.0029309)
Supplement: Table S2 — Primer sequences used for the sequence analysis of OPN variants. (DOC) [file pone.0029309.s002.doc]

###### Supplemental Table S2

| **Polymorphism** | **Primer sequences** |
| --- | --- |
| rs2728127 | GGCTCCAGCATAATCTATTCCTA  TGGTTTGTAGGTAATAAGCACCCAT |
| rs2853744 | CATGGATGAGGGAACAAGGATA  AACCACTCTTGCCTGTATGATTGTA |
| rs11730582 | CATGGATGAGGGAACAAGGATA  AACCACTCTTGCCTGTATGATTGTA |
| rs11739060 | GCTAGTTAATGATATTGTACATAAGTAA  TGACAACCAAGCCCTCCCAGAA |
| rs28357094 | CTGAATGCCCATCCCGTA AAGCCCTCCCAGAATTTAAATGC |
| rs4754=p.Asp80Asp | TAATTTTCAGACCCTTCCAAG  GTGAGACTCATCAGACTGGTGAGAA |
| rs1126616=p.Ala236Ala | TGAAACCCACAGCCACAAG  ATGGCTGTGGAATTCACGG |
| rs1126772 | CATGAATTAGATAGTGCATCTTCTGAGG  TGTTATATTCTCTTTTTAAGTGGGTA |
| rs9138 | CATGAATTAGATAGTGCATCTTCTGAGG  TGTTATATTCTCTTTTTAAGTGGGTA |

**Supplemental Table S2. Primer sequences used for the sequence analysis of *OPN* variants.**
